# Temporal changes in monocytic and neutrophilic trem-1 and trem-2 surface receptors in blood and bronchoalveolar lavage fluid in the development and resolution of ventilator-associated pneumonia (vap)

**DOI:** 10.1186/2197-425X-3-S1-A434

**Published:** 2015-10-01

**Authors:** V Grover, P Kelleher, S Singh

**Affiliations:** Royal Marsden NHS Foundation Trust, Critical Care, London, United Kingdom; Magill Department of Critical Care, Anaesthesia and Pain, Chelsea and Westminster Hospital NHS Foundation Trust, London, United Kingdom; Department of Surgery and Cancer, Imperial College London, London, United Kingdom; Immunology Section, Department of Medicine, Imperial College London, London, United Kingdom; Department of Immunology, Imperial College Healthcare NHS Trust, London, United Kingdom; Department of Respiratory Medicine, Chelsea and Westminster Hospital NHS Foundation Trust, London, United Kingdom

## Introduction

TREM-1 (Triggering Receptor Expressed on Myeloid Cells) amplifies Toll-like receptor (TLR) responses and is a putative biomarker to diagnose VAP. It exists on the surface of monocytes/macrophages (mTREM-1) and neutrophils (nTREM-1) as well as a soluble receptor (sTREM-1). Standalone levels of mTREM-1 in bronchoalveolar lavage fluid (BALF) and its BALF/blood ratio are elevated in VAP (1). TREM-2 (a negative TLR regulator) may also have a role in pneumonia (2), but there is a paucity of data in humans.

## Objectives

To measure temporal changes in monocytic/macrophage and neutrophilic TREM-1 and TREM-2 surface receptor levels in blood and BALF during VAP development and resolution.

## Methods

Serial receptor levels were assessed in 12 patients. 5 had levels measured prior to development of VAP, 2 with clinical worsening of VAP, 6 with VAP resolution and in one patient changes were tracked in all three stages. Patients were recruited in a teaching hospital ICU (mixed medical, surgical and burns patients). Alternate day phlebotomy and bronchoscopy were performed. TREM-1 (12 patients) and TREM-2 levels (6 patients) on neutrophils and monocytes/macrophages in blood and BALF were assessed using flow-cytometry. VAP was diagnosed using the clinical pulmonary infection score (CPIS) and semi-quantitative microbiology and confirmed using HELICS criteria. In addition, peripheral blood white cell count and CRP levels were determined.

## Results

mTREM-1 in BALF and its BALF/blood ratio rose in four out of the five patients each prior to VAP development (static in one out of five). BALF mTREM-1 and its BALF/blood ratio rose in both patients with worsening VAP and fell in five out of six patients with resolving VAP (static in the other). White cell count and CRP levels did not correlate with VAP.

Neutrophilic TREM-1 changes in BALF broadly fitted two patterns: either mirroring changes with mTREM-1 but with a lower magnitude, or falling with infection development.

Considering TREM-2, BALF mTREM-2 either remained similar or rose prior to VAP development of VAP. The level rose with worsening VAP and fell with resolution, akin to the changes in mTREM-1. For nTREM-2, levels remained static prior to infection, but rose during VAP development and resolution.

## Conclusions

BALF mTREM-1 and its BALF/blood ratio rise and fall with VAP development and resolution, highlighting the potential role of TREM-1 in the pulmonary immune response and its role as a putative biomarker for VAP diagnosis. Monocyte and neutrophils differed in their changes in TREM-1 and TREM-2 receptor levels over time. TREM-1 and TREM-2 warrant further study in patients with pneumonia.

GRANT ACKNOWLEDGEMENT

This study was funded by the National Institute of Academic Anaesthesia and the Westminster Medical School Trust Fund
